# Dehiscent fruits in Brassicaceae and Papaveraceae: convergent morpho-anatomical features with divergent underlying genetic mechanisms

**DOI:** 10.1093/aob/mcaf079

**Published:** 2025-05-04

**Authors:** Cecilia Zumajo-Cardona, Barbara A Ambrose, Yesenia Madrigal, Natalia Pabón-Mora

**Affiliations:** Botanical Garden, University of Padova, Padova, Italy; Department of Biology, University of Padova, Italy; The New York Botanical Garden, New York, USA; The New York Botanical Garden, New York, USA; Universidad de Antioquia, Medellín, Colombia; Universidad de Antioquia, Medellín, Colombia

**Keywords:** Dehiscence zone, dry fruits, fruit development, fruit patterning, Papaveraceae

## Abstract

**Background and Aims:**

Dry dehiscent fruits have independently evolved multiple times during angiosperm diversification. A striking example is the convergent evolution of Brassicaceae siliques and Papaveraceae pods, both formed by two fused carpels forming valves that meet at a replum or replum-like structure. In both cases, valve separation occurs through a dehiscence zone at the valve margins in contact with the replum. In *Arabidopsis*, fruit development is regulated by transcription factors: *FRUITFULL* (*FUL*) ensures proper valve cell division, *REPLUMLESS* (*RPL*) specifies replum identity and *SHATTERPROOF* (*SHP1*/*2*) genes pattern the dehiscence zone. *SHP1/2* also regulate *INDEHISCENT* (*IND*) for lignified layer formation and *ALCATRAZ* (*ALC*) and *SPATULA* (*SPT*) for the non-lignified layer. The network is downregulated by *APETALA2* (*AP2*), which influences replum formation and valve margin growth.

**Methods:**

Using previously published and new *in situ* RNA hybridization expression data, we evaluated how this network applies to basal eudicots.

**Key Results:**

In *Bocconia frutescens*, homologue expression suggests conserved roles for *FUL* and *AP2* in fruit wall proliferation, acting antagonistically to *ALC* and *RPL* homologues localized to the dehiscence zone. A role for *STK* homologues in dehiscence zone formation cannot be excluded, while a role of *AG-like* genes, the closest homologues of *SHP* during fruit development, is unlikely.

**Conclusions:**

Our findings indicate significant rewiring of the fruit developmental network between basal and core eudicots, underscoring the need for functional studies in non-eudicot species to validate this framework.

## INTRODUCTION

Fruit dehiscence is the process where the fruit wall ruptures to release seeds. This process can vary greatly in its anatomical and positional features. Roth (1977; see also Zimmerman, 1959) identified two key mechanisms that can occur in dehiscent fruits during maturation: (1) separation among sutures, which are areas of posgenital fusion during carpel development such as the carpel margins (i.e. ventricidal or septicidal); or 2) separation inside the carpel wall itself (i.e. loculicidal, poricidal or circumscissile). In addition, dehiscence can be triggered by either cell rupturing (often less specialized) or cell separation (more specialized; see Bobrov and Romanov, 2019 and references therein). The anatomical changes that lead to dehiscence can involve various factors, such as the arrangement and size of similar cell types, variations in cell turgor, or the opposing arrangement of tissues that create tension and weak spots (Haberlandt, 1924; Guttenberg, 1971; Roth, 1977; Bobrov and Romanov, 2019). Ultimately, differences in how cells swell and shrink during development generate the tension needed to rupture the fruit and release seeds.

One of the most studied anatomical variations related to fruit dehiscence is when one or more layers of lignified cells come in direct contact with parenchymatic cells. During maturation, these parenchymatic cells undergo changes in turgor and tension. This phenomenon is frequently observed in model plants, particularly in Fabaceae pods (Christiansen *et al.*, 2002; Wang and Grusak, 2005; Fourquin *et al.*, 2013; Aguilar-Benitez *et al.*, 2020; Cerri and Reale, 2020; DiVittori *et al.*, 2021), Solanaceae capsules (Pabón-Mora and Litt, 2011; Ballester and Ferrándiz, 2017) and Brassicaceae siliques (Spence *et al.*, 1995; Davies and Bruce, 1997; Ferrándiz, 2002; Liljegren *et al.*, 2004). Typically, in these taxa, a parenchymatic mesocarp surrounds either a fibrous vascular bundle (common in legumes) or a lignified endocarp (as seen in Brassicaceae and Solanaceae). This arrangement of sclerenchymatous and parenchymatous tissues side by side is also common outside eudicots, for instance in the follicles of some early angiosperm ANA lineages (e.g. *Illicium* in Austrobaileyales; Romanov *et al.*, 2013), Magnoliids (e.g. *Saruma* in Piperales and *Magnolia* in Magnoliales; Bobrov and Romanov, 2019) and basal eudicots (e.g. *Aquilegia* in Ranunculales; RanOmics, 2024). Additionally, some variations can lead to explosive dehiscence. For example, in *Cardamine hirsuta*, asymmetric lignin deposition in the endocarp cells, along with precise microtubule-dependent growth in surrounding cell layers, causes explosive opening (Hofhuis *et al.*, 2016; Cullen and Hay, 2024). This highlights that tissue specialization and organization can vary slightly, yet result in convergent dry dehiscent fruits.

Fruit patterning is controlled by several transcription factors and downstream hormones, which have been best studied in core-eudicot model species. For example, *Arabidopsis thaliana* features a syncarpous bicarpellate gynoecium, which during fruit development transitions into two lateral valves and a median persistent replum that connects to the septum. During fruit opening, the valves separate from the replum through the marginal dehiscence zone. In this species fruit development is controlled by *FRUITFULL* (*FUL*), which promotes cell proliferation in the fruit wall (Gu *et al.*, 1998; Ferrándiz, 2002), and *REPLUMLESS* (*RPL*), which controls replum development (Roeder *et al.*, 2003; Roeder and Yanofsky, 2006). *FUL* and *RPL* negatively repress *SHATTERPROOF 1* (*SHP1*) and *SHP2*, which act as key factors in the formation of the dehiscence zone in the valve margins (i.e. between the valves and the replum; Liljegren *et al.*, 2000; Roeder and Yanofsky, 2006). *SHP* genes redundantly upregulate two additional transcription factors, *INDEHISCENT* (*IND*) and *ALCATRAZ* (*ALC*), which promote different cell fates inside the dehiscence zone, controlling the formation of a lignified layer (*IND*) and a separate parenchymatic layer (both *IND* and *ALC*; Rajani and Sundaresan, 2001; Liljegren *et al.*, 2004). Later, *APETALA2* (*AP2*) was included in the core regulators of fruit development, as a negative regulator of both *RPL* and *SHP*, as it was found to prevent overgrowth of both the replum and the valve margin (Ripoll *et al.*, 2011). Because these genes control to some extent histological organization and tissue patterning in the valves, the valve margins and the replum, changes in their expression can be linked to shifts in dehiscence patterns, at least in Brassicaceae (Mummenhoff *et al.*, 2009; Avino *et al.*, 2012).

All genes in this network belong to different gene families. *FUL* and *SHP* are MADS-box MIKCc transcription factors, *RPL* is a homeodomain gene, *IND* and *ALC* are members of the bHLH (basic helix loop helix) gene family, and *AP2* is part of the AP2/ETHYLENE RESPONSIVE FACTOR (ERF) family (revised in Pabón-Mora *et al.*, 2014). One issue that arises when examining how this genetic regulatory network influences dehiscent fruit development in non-core eudicots is the lack of corresponding orthologues for these genes outside of core eudicots. This is so because most of these gene families have duplicated and functionally diversified concomitant with the evolution of core eudicots (Pabón-Mora *et al.*, 2014). For instance, *FUL* is one of four paralogues of the *AP1/FUL* gene lineage in *Arabidopsis*, most core eudicots have *FUL* orthologues, but basal eudicots only have *FUL-like* pre-duplication genes (Litt and Irish, 2003). Similarly, *SHP1* and *SHP2* are Brassicaceae paralogues, and while other eudicots do have *SHP* orthologues, the basal eudicots only have pre-duplication *AGAMOUS-like* genes (Kramer *et al.*, 2004). *ALC* orthologues are present across core eudicots and, by comparison, basal eudicots only have the pre-duplication palaeo *SPT/ALC* genes (Pabón-Mora *et al.*, 2014). Finally, *INDEHISCENT* is only present in Brassicales as a result of a local duplication, while other core and basal eudicots have genes more similar to its paralogue *HECATE3* (Pabón-Mora *et al.*, 2014).

In this paper we aim to: (1) clarify which homologues of the fruit genetic regulatory network are present in basal eudicots when compared to model core eudicots in the context of the evolution of each gene lineage; (2) summarize their expression patterns during fruit patterning in the dry dehiscent fruit of *Bocconia frutescens* (Papaveraceae), a basal eudicot; (3) hypothesize their putative roles in the patterning of these dry dehiscent fruits based on expression data; and (4) assess if rewiring of the fruit patterning genes has occurred in the transition from basal to core eudicots.

## MATERIALS AND METHODS

### Phylogenetic analyses

Homologues of the different gene lineages were obtained based on previous studies (Pabón-Mora *et al.*, 2014; Zumajo-Cardona *et al.*, 2017, 2018, 2021; Supplementary Data Table S1). Additionally, the BLAST search was extended to more species belonging to the Ranunculales (including homologues from Berberidaceae, Papaveraceae, Eupteleaceae, Lardizabalaceae, Menispermaceae and Ranunculaceae). Databases used include OneKP (https://db.cngb.org/onekp/) and PhytoMetaSyn (Xiao *et al.*, 2013). The phylogenetic hypotheses were done by maximum likelihood (ML) using the desktop version of IQ-TREE software (http://www.iqtree.org; Nguyen *et al.*, 2015; Minh *et al.*, 2020). The molecular evolution model that best fits to the data was found with ModelFinder on IQ-TREE (Kalyaanamoorthy *et al.*, 2017). Branch support was calculated with ultrafast bootstrap (UFBS) of 1000 pseudo-replicas, also available in IQ-TREE (Hoang *et al.*, 2018). The trees obtained were observed using FigTree v.1.4.4 (http://tree.bio.ed.ac.uk/software/figtree/).

### In situ hybridization

To detect the expression patterns of specific genes through *in situ* hybridization we have collected and processed *B. frutescens* plant material as previously described (Zumajo-Cardona *et al.*, 2017, 2018, 2021). For probe synthesis of the *FUL-like* and *AG-like* homologues (*BofrFL1,2,3* and *BofrAG*) we designed specific primers for each gene (Supplementary Data Fig. S1). The *in situ* hybridization protocol, including the steps of probe synthesis, was performed as previously described in Ambrose *et al.* (2000).

## RESULTS

### Phylogenetic analyses of the canonical fruit network genes

#### APETALA2 (AP2) genes.

**Fig. 1.**
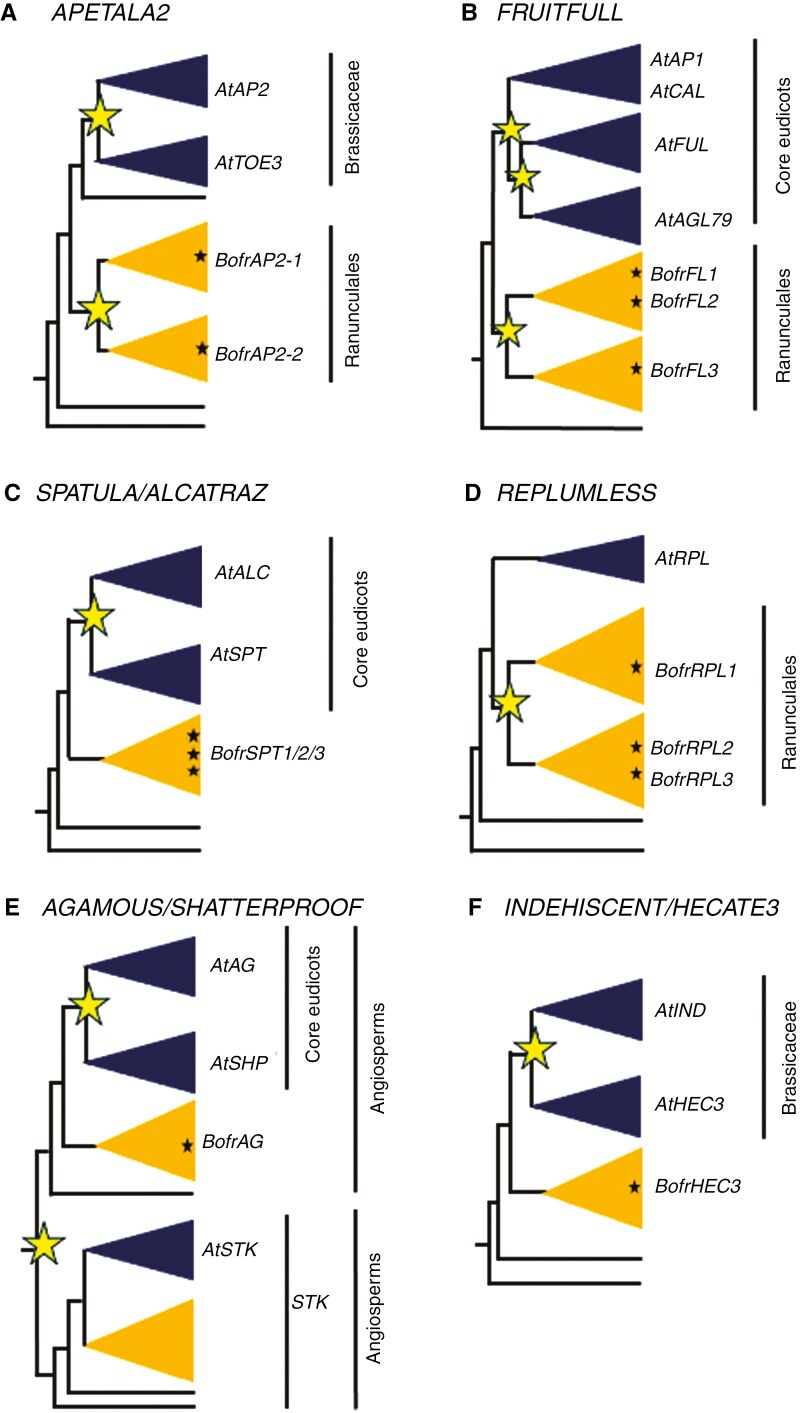
Schematic representation of the evolutionary history of the different gene lineages that form the fruit developmental network in *Arabidopsis*. (A) *APETALA2* gene lineage. (B) *FRUITFULL* gene lineage. (C) *SPATULA/ALCATRAZ* gene lineage. (D) *REPLUMLESS* gene lineage. (E) *AGAMOUS/SHATTERPROOF* gene lineage. (F) *INDEHISCENT/HECATE3* gene lineage. Several independent duplication events have been identified; here the yellow stars highlight duplication events that have occurred in the Brassicaceae, core eudicots and Ranunculales. In dark blue are the Brassicaceae or core eudicot clades, and in yellow the gene clades belonging to Ranunculales. *Bofr*: genes belonging to *Bocconia frutescens*.

*AP2* belongs to the *AP2/ETHYLENE RESPONSIVE FACTOR* (*ERF*) gene lineage, a large transcription factor family present across Viridiplantae (Bowman *et al.*, 1989; Jofuku *et al.*, 1994; Elliott *et al.*, 1996; Moose and Sisco, 1996). Within this gene lineage is the *euAP2* clade, which is present across vascular plants (Kim *et al.*, 2006; Zumajo-Cardona and Pabón-Mora, 2016); this clade contains the subclade *AP2/TARGET OF EAT3* (*TOE3*) to which the *AP2* homologue of Arabidopsis belongs (Kim *et al.*, 2006). In that subclade, the two Arabidopsis paralogues, *AP2* and *TOE3*, are the result of a Brassicaceae-specific duplication event (Fig. 1A; Supplementary Data Fig. S2). Pre-duplication homologues are present across vascular plants, and they are more similar to AP2 than to TOE3 in terms of sequence identity (Zumajo-Cardona *et al.*, 2021). Several other local duplication events have been identified in Solanaceae, monocots and basal eudicots. In the basal eudicots we have confirmed a duplication event occurring early on in the diversification of the order Ranunculales, perhaps after the diversification of Eupteleaceae. Thus, most members of Ranunuculales have two AP2 paralogues. Only in Lardizabalaceae were no *AP2* homologues retrieved (Fig. S2). Within the Ranunculales, taxon-specific duplication events were identified in *Capnoides sempervirens*, *Ceratocapnos vesicaria*, *Corydalis chelanthifolia*, *Nandina domestica*, *Papaver bracteatum*, *Papaver rhoeas*, *Papaver setigerum*, *Papaver somniferum* and *Xanthorhiza simplicissima* (Fig. S2).

In *Bocconia frutescens* there are two paralogues, *BofrAP2-1*, each in a separate Ranunculales clade (Fig. 1A).

#### FRUITFULL-like (FUL-like) genes.

*FUL* is one of four *Arabidopsis* paralogues in the *APETALA1/FRUITFULL* gene lineage, belonging to the MADS-box gene family (Purugganan *et al.*, 1995; Gu *et al.*, 1998; Becker and Theissen, 2003). Two duplication events occurred prior to the diversification of core eudicots giving rise to the *euFULI* (containing the Arabidopsis *FUL*), *euFULII* (which includes the Arabidopsis *AGL79*) and *euAP1* lineages. The last of these has undergone an additional duplication event specific to Brassicaceae (resulting in the Arabidopsis paralogues *CAL* and *AP1*; Fig. 1B; Supplementary Data Fig. S3) (Litt and Irish, 2003). Outside core eudicots, additional large-scale duplications have been detected, for example in the Ranunculales (Fig. 1B; Fig. S3; Pabón-Mora *et al.*, 2013a). Additionally, several taxon-specific duplication events have been identified in *Argemone mexicana*, *Aquilegia coerulea*, *Berberis gilgiana*, *Bocconia frutescens*, *Eschscholzia californica*, *Eranthis hyemalis*, *Macleaya cordata*, *Pseudofumaria lutea* and *Papaver setigerum*.

In *Bocconia frutescens* there are three paralogues, *BofrFL1* and *BofrFL2* in one clade, and *BofrFL3* in another clade (Fig. 1B).

#### SPATULA (SPT)/ALCATRAZ (ALC) genes.

*SPT* and ALC genes belong to the bHLH superfamily of transcription factors present in animals and plants (Atchley *et al.*, 1997; Lendent and Vervoort, 2001; Toledo-Ortiz *et al.*, 2003; Groszmann *et al.*, 2008; Pires and Dolan, 2010). The *SPT* and *ALC* paralogues from *Arabidopsis* are the result of a duplication event prior to the diversification of core eudicots (Pabón-Mora *et al.*, 2014; Zumajo-Cardona *et al.*, 2017; Fig. 1C; Supplementary Data Fig. S4). With the extended sampling in Ranunculales, only one clade of *paleoSPT/ALC* was detected. This lineage has not undergone major duplication events outside core eudicots. However, several taxon-specific duplications were found in *Akebia quinata*, *Akebia trifoliata*, *Bocconia frutescens*, *Capnoides sempervirens*, *Hydrastis canadensis*, *Jeffersonia diphylla*, *Papaver bracteatum*, *Papaver rhoeas*, *Papaver setigerum* and *Papaver somniferum*.

In *Bocconia frutescens* there are three paralogues, *BofrSPT1*, *BofrSPT2* and *BofrSPT3*, as a result of species-specific duplications (Fig. 1C).

#### REPLUMLESS (RPL) genes.

*RPL* is a homeodomain protein, specifically belonging to the TALE class that contains a ZIBEL motif (Bürglin, 1997; Becker *et al.*, 2002; Kumar *et al.*, 2007; Mukherjee *et al.*, 2009). The *RPL* clade is the result of a duplication event prior to the diversification of all angiosperms that also gave rise to the *POUND FOOLISH* (*PNF*) clade (Pabón-Mora *et al.*, 2014). The *RPL* lineage has undergone one duplication event before the diversification of Ranunculales (Fig. 1D; Supplementary Data Fig. S5). In addition, some taxon-specific duplication events were identified in *Akebia trifoliata*, *Bocconia frutescens*, *Cissampelos mucronata*, *Corydalis cheilanthifolia*, *Papaver bracteatum* and *Papaver rhoeas.*

In *Bocconia frutescens* there are three paralogues, *BofrRPL1* in one clade and *BofrRPL2* and *BofrRPL3* in another clade (Fig. 1D).

#### SHATTERPROOF (SHP) genes.

The two SHP genes (*SHP1* and *SHP2*) present in *Arabidopsis* are the result of a duplication event specific to Brassicales. All other core eudicots have *SHP* and *AGAMOUS* homologues, as the result of a duplication event prior to the diversification of core eudicots (Kramer *et al.*, 2004; Dreni *et al.*, 2013; Pabón-Mora *et al.*, 2014). Pre-duplication genes outside core eudicots are called paleoAGAMOUS as their protein sequence resembles that of AGAMOUS (Kramer *et al.*, 2004; Pabón-Mora *et al.*, 2014). Hence, all Ranunculales homologues belong to the *paleoAGAMOUS* clade (Fig. 1E), where no major duplication events have been identified (Supplementary Fig. S6). Taxon-specific duplication events have been detected within Ranunculales in *Argemone mexicana*, *Eschscholzia californica*, *Euptelea pleiosperma* and *Thalictrum thalictroides.*

In *Bocconia frutescens* there is a single *AG-like* (or *paleoAG*) homologue, *BofrAG* (Fig. 1E).

#### INDEHISCENT (IND) / HECATE3 (HEC3) genes.

*IND/HEC3* are two bHLH genes (Heim *et al.*, 2003; Toledo-Ortiz *et al.*, 2003). *IND* and *HEC3* are the result of a duplication event specific to Brassicales, and all pre-duplication genes, most of them single-copy, appear to be more similar in sequence to *HEC3* than to *IND* (Kay *et al.*, 2013; Pabón-Mora *et al.*, 2014; Fig. 1F). In Ranunculales *HEC3-like* homologues remain single copy with a few exceptions such as in *Eschscholzia californica*, *Papaver setigerum*, *Papaver rhoeas* and *Papaver somniferum* (Supplementary Fig. S7).

In *Bocconia frutescens* there is a single *HEC3-like* homologue, *BofrHEC3* (Fig. 1F).

### Expression of fruit development transcription factors in Bocconia frutescens

**Fig. 2.**
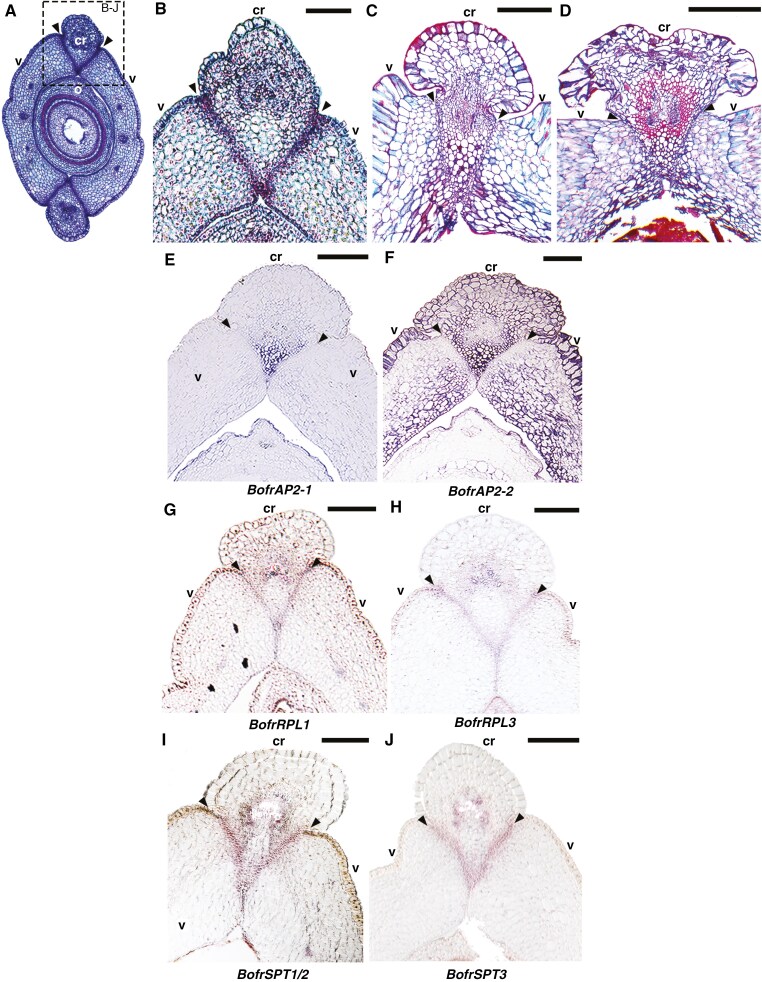
*Bocconia frutescens* carpel to fruit transition and summary of *in situ* hybridization expression patterns of *AP2*, *RPL* and *SPT* homologues following Zumajo-Cardona *et al*. (2017, 2018, 2021). (A) Cross-section through a carpel at anthesis. Inset points to detail of the dehiscence zones between the valves and the commissural ring shown in all other panels (B–J). (B–D) Detail of the fruit dehiscent zone at three different continuous stages of fruit development. Note the increase in lignification around the vasculature of the commissural ring and the smaller, thinner cells forming the dehiscence zone. (E–F) Expression patterns of *APETALA2* homologues in *B. frutescens* fruits. Note lack of expression in the dehiscence zone. (G–H) Expression patterns of *REPLUMLESS* homologues. Note restricted expression to the dehiscence zone. (I–J) Expression patterns of *SPATULA/ALCATRAZ* homologues. Note overlapping expression with RPL genes to the dehiscence zone. Arrowheads point to the dehiscence zone; cr, comissural ring; o, ovule; v, valve. Scale bars: 200 µm (B, E–J), 500 µm (C, D).

Previous studies have reported the expression of *BofrAP2*, *BofrSPT* and *BofrRPL* homologues (Zumajo-Cardona *et al.*, 2017, 2018, 2021). *BofrAP2-1* is only expressed in the commissural ring, while *BofrAP2-2* is present in both the commissural ring and the valves (Fig. 2; Zumajo-Cardona *et al.*, 2021). All three *RPL* homologues, *BofrRPL1*, *BofrRPL2* and *BofrRPL3*, are restricted to the dehiscence zone (Zumajo-Cardona *et al.*, 2018). Similarly, *BofrSPT1/2* and *BofrSPT3* were all found expressed in the dehiscence zone, between the valves and the commissural ring (Fig. 2; Zumajo-Cardona *et al.*, 2017). However, expression of *BofrFL1-3*, the *FUL-like* genes, the closest *FRUITFULL* homologues, and *BofrAG*, the *AG-like* gene, the closest *SHATTERPROOF* homologue, had not previously been assessed in *Bocconia frutescens.*

**Fig. 3.**
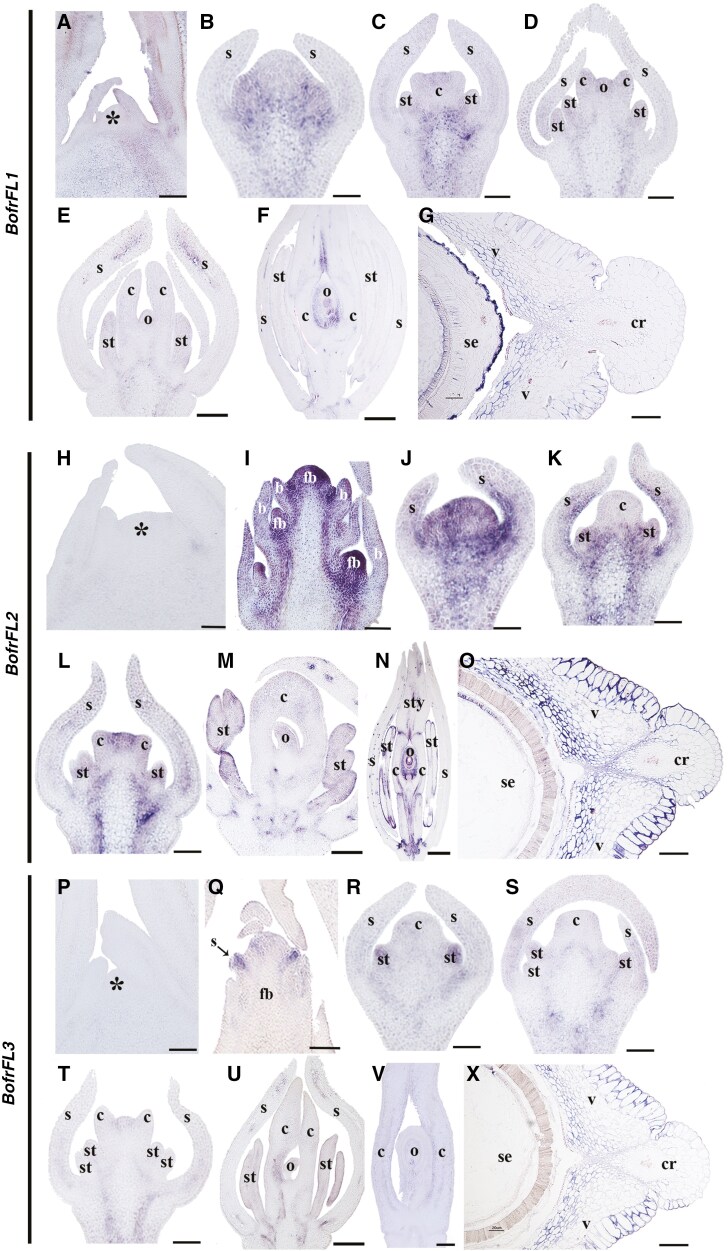
*In situ* hybridization expression patterns of *FUL-like* homologues in *Bocconia frutescens.* Expression of *BofrFL1* in longitudinal (A–F) and cross (G) sections of developing shoots, flower and fruits. (A) Shoot apical meristem and flanking leaves. (B) Floral bud in stage 3. Note *BofrFL1* expression in the developing organs in the meristem. (C–E) Floral development stages 4–6. (F) Floral development stage 8. Note persistent *BofrFL1* expression in the ovule and the style transmitting tissue. (G) Fruit. Note no *BofrFL1* expression. Expression of *BofrFL2* in longitudinal (H–N) and cross (O) sections of developing shoots, flowers and fruits. (H) Shoot apical meristem and flanking leaves. (I) Young inflorescence with floral buds in stages 1–3. Note *BofrFL2* expression in all developing floral buds. (J–L) Floral development stages 3–5. Note *BofrFL2* expression in stamens and carpels. (M-N) Floral development stages 6 and 8. Note persistent *BofrFL2* expression in the junctions between gynophore, ovary and style, as well as in the ovule. (O) Fruit. Note expression of *BofrFL2* in the valve and the commissural ring. Expression of *BofrFL3* in longitudinal (P–V) and cross (X) sections of developing shoots, flowers and fruits. (P) Shoot apical meristem and flanking leaves. (Q) Floral bud in stage 2. Note *BofrFL3* expression in the sepals. (R–U) Floral development stages 4–6. (V) Floral development stage 8. Note no *BofrFL3* expression. (X) Fruit. Note *BofrFL3* expression in the valves. Asterisks indicate the shoot apical meristem; b, bract; c, carpel; cr, commissural ring; o, ovule; s, sepal; se, seed; st, stamen; sty, style; v, valve. Scale bars: 50 µm (A–D, H–L, P, R–T), 100 µm (E, F, I, M, N, Q, U, V), 250 µm (G, O, X).

Here we detected expression of only *BofrFL1* in shoot apical meristems (Fig. 3A, H, P). All three paralogues are active in floral meristems, and during the formation of all floral organs, especially *BofrFL2* (Fig. 3B–D, I–L, R–T). Expression of *BofrFL3* seems to be predominantly restricted to the stamens (Fig. 3R–S). All three copies are expressed during carpel patterning and in the ovule, but expression of *BofrFL2* is restricted to the junctions between the gynophore and the ovary, as well as between the ovary and the style (Fig. 3N). Finally, only *BofrFL2* and *BofrFL3* are expressed during fruit development (Fig. 3G, O, X). While *BofrFL2* is strongly expressed in the valves and the commissural ring (Fig. 3O), *BofrFL3* is expressed in the valves (Fig. 3X).

**Fig. 4.**
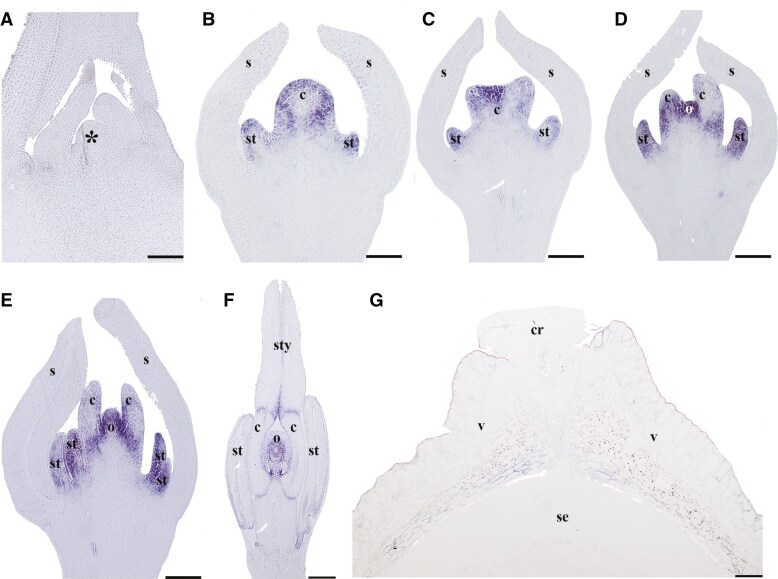
*In situ* hybridization expression patterns of *AG-like* homologues in *Bocconia frutescens* in longitudinal (A–F) and cross (G) sections of developing shoots, flowers and fruits. (A) Shoot apical meristem and flanking leaves. (B) Floral bud in stage 3. Note *BofrAG* expression in stamens and carpels. (C–E) Floral development stages 4–6. Note maintained expression of BofrAG in stamens, carpels and the developing ovule. (F) Floral development stage 8. Note persistent *BofrAG* expression in the ovule and the style transmitting tissue. (G) Fruit. Note no *BofrAG* expression. Asterisk indicates the shoot apical meristem; c, carpel; cr, commissural ring; o, ovule; s, sepal; se, seed; st, stamen; sty, style; v, valve. Scale bars: 50 µm (A–E), 100 µm (F), 250 µm (G).

The homologue of *AGAMOUS* in *B. frutescens*, *BofrAG*, is only expressed at late floral development stages during the initiation of stamens, carpels and ovules (Fig. 4A–E). Late in flower development, *BofrAG* is also expressed in the junction between the gynophore and the ovary, as well as in the junction between ovary and style, and in the developing ovule (Fig. 4F). *BofrAG* is not expressed during fruit development (Fig. 4G).

## DISCUSSION

**Fig. 5.**
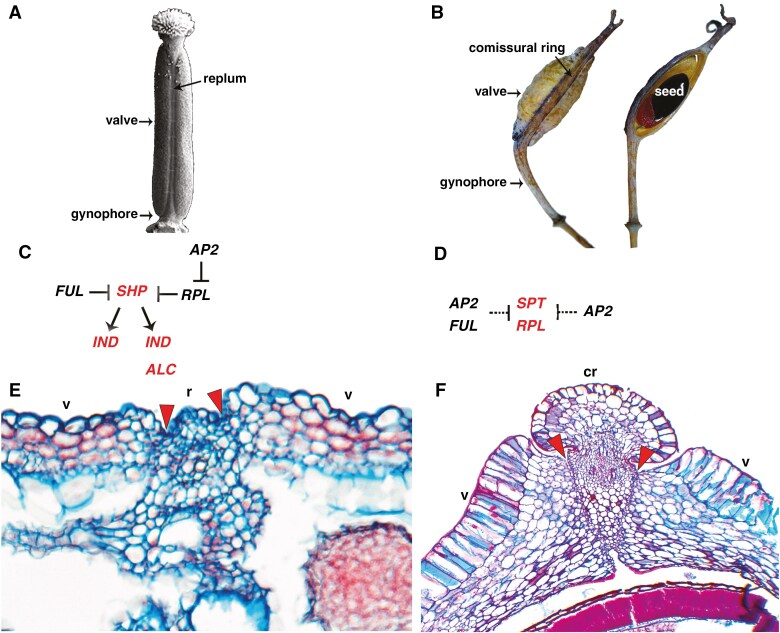
Comparison between the fruit genetic networks in *Arabidopsis thaliana* (Brassicaceae) (A) and *Bocconia frutescens* (Papaveraceae) (B). (C) Putative genetic players in *A. thaliana*. In black are those important for valve and replum development, in red are those associated with the dehiscence zone. In *A. thaliana FRUITFULL* (*FUL*) is associated with valve development and *REPLUMLESS* (*RPL*) controls replum development. The two negatively regulate *SHATTERPROOF* (*SHP*) restricting its expression to the dehiscence zone. *SHP* activates *ALCATRAZ* and *INDEHISCENT* to shape the parenchymatic or separation layer and the lignified layer, respectively. Finally, *APETALA2* (*AP2*) seems to downregulate *RPL* during fruit patterning. (D) Putative genetic players in *B. frutescens*. Here. there are no true orthologues of *SHP* and *IND*, and homologues such as A*GAMOUS-like* (*AG-like*) and *HECATE3* (*HEC3*) respectively, are not actively expressed in fruit development. In this case *AP2* and *FUL* are restricted to valves, while only *AP2* seems to act in the commissural ring, the replum-like structure. Conversely, *SPATULA* (*SPT*) and *RPL* are restricted to the dehiscence zone. (E) Cross-section of *A. thaliana* fruit. (F) Cross-section of *B. frutescens* fruit. Red arrowheads indicate dehiscence zones. cr, commissural ring; r, replum; v, valve.

Convergent evolution in fruit types is extremely frequent across angiosperms. Dry dehiscent fruits, for instance, have occurred multiple times during evolution releasing the seeds after rupturing of the pericarp. One such case can be observed in Brassicaceae and Papaveraceae, both having: superior ovaries formed by two carpels congenitally fused, a gynophore, a medial replum or replum-like structure located between the valves, and dehiscence zones between the valve margins and the replum (or replum-like tissue; Roeder and Yanofsky, 2006) (Fig. 5). Moreover, Brassicaceae is a rosid in the core eudicots, while Papaveraceae occupies a phylogenetic position in the Ranunculales in the basal eudicots. As many transcription factors have duplicated concomitant with the evolution of core eudicots, this comparison between Brassicaceae and Papaveraceae fruits allows us to evaluate genetic hubs retained and those that have been rewired in the two families to construct convergent dry dehiscent fruits.

Based on our current study and previous findings, we conclude that both *FRUITFULL* orthologues and pre-duplication *FUL-like* genes play crucial roles in fruit development (reviewed in Ferrándiz and Fourquin, 2014). These genes are actively expressed in the carpel wall and fruit valves (Fig. 3; Pabón-Mora *et al.*, 2012). In both Brassicaceae and Papaveraceae, downregulation of *FUL* homologues leads to premature rupture of the fruit wall and significant defects in cell proliferation, particularly in the endocarp (Gu *et al.*, 1998, Pabón-Mora *et al.*, 2012, 2013*a*). Interestingly, *FUL-like* genes may not play significant roles in other basal eudicots; for instance, downregulation of *FUL-like* genes does not affect fruit patterning in the follicles of *Aquilegia* (Pabón-Mora *et al.*, 2013*b*).

Another key transcription factor that seems to retain fruit development roles in core and basal eudicots is *APETALA2*. During fruit development, *AP2* in *Arabidopsis* controls replum growth and valve margin formation by a direct negative regulation of *SHATTERPROOF*, *BREVIPEDICELLUS* and *REPLUMLESS* (Ripoll *et al.*, 2011). In *Bocconia frutescens* the two local *AP2* copies show overlapping expression only in the commissural tissue and one of the homologues is also expressed in the fruit wall. Notably, both copies are absent from the dehiscence zone (Fig. 2). Similar expression patterns restricted to the fruit wall and absent from the future deshiscence zones have been documented in the fruit wall of *Aristolochia fimbriata* (a magnoliid) (Zumajo-Cardona *et al.*, 2021). These expression patterns suggest a role for *AP2* genes in fruit wall development, probably acting as repressors of dehiscence zone-specific genes, across angiosperms.

Conversely, the available data suggest that *SHATTERPROOF* orthologues and pre-duplication *AG-like* genes contribute differently to fruit patterning (reviewed in Ferrándiz and Fourquin, 2014). *SHP* genes are found exclusively in core eudicots, where they play pivotal roles in fruit patterning, particularly in defining dehiscence zone identity in Brassicaceae (Liljegren *et al.*, 2000). In contrast, *AG-like* genes in species such as *Eschscholzia californica* and *Papaver somniferum* are expressed in stamens and carpels, where they regulate organ identity, functioning similarly to *AGAMOUS* in *Arabidopsis* (Yellina *et al.*, 2010; Hands *et al.*, 2011). The role of *AG-like* genes in controlling stamen and carpel identity, along with the closure of the floral meristem, is conserved in other Ranunculales, including *Nigella* (Wang *et al.*, 2015) and *Thalictrum* (Galimba *et al.*, 2012). Downregulation of *AG-like* genes in these species results in petal proliferation and floral indeterminacy, with the absence of stamens and carpels. This raises questions about whether *AG-like* genes contribute to fruit development. However, the lack of *AG-like* gene expression in fruits suggests they may not play a critical role in forming the dehiscence zone during fruit patterning, as with their *SHP* counterparts in Brassicales.

This scenario suggests that basal eudicots deployed different genes for defining the dehiscence zone before the evolution of *SHP* genes. For example, the closely related MADS-box D-class *SEEDSTICK* (*STK*) homologues are critical for fruit patterning in *Eschscholzia californica* (Lotz *et al.*, 2024) as they repress valve tissue proliferation. In addition, *STK* genes are expressed in the dehiscence zone of dry dehiscent fruits in *Aristolochia fimbriata* (Suárez-Baron *et al.*, 2017). In *Arabidopsis*, *STK* also regulates tissue degradation processes, such as seed abscission (Balanzá *et al.*, 2016), suggesting that retained roles of *STK* may include patterning of dehiscence zones and abscission.

Other candidate genes for dehiscence zone identity include *SPT* and *RPL* homologues, which are specifically expressed in the dehiscence zone during fruit differentiation. In *Bocconia frutescens*, *SPT* genes are expressed in the two or three cell layers forming the dehiscence zone, while expression of *RPL* genes seems more restricted to a single cell layer, probably the first to degrade (Fig. 2). The roles of *SPT* and *RPL* homologues in tissue specification during dehiscence and/or abscission have been previously documented. For instance, in *Arabidopsis*, *SPT* and its core eudicot paralogue *ALC* are essential for retaining parenchymatic identity within the separation layer of the dehiscence zone, a key factor for fruit opening (Rajani and Sundaresan, 2001; Girin *et al.*, 2011). Similarly, the *RPL* homologue *qSH1* in rice controls grain abscission. In domesticated rice varieties, loss of shattering is linked to mutations in *qSH1* (Konishi *et al.*, 2006). These findings suggest that *SPT*, *STK* and *RPL* genes probably played critical roles in forming the separation layer between the valves of the dehiscence zone in early-diverging angiosperms and basal eudicots, well before the evolution of *SHP* genes.

Other putative key players of the dehiscence zone include *IND* homologues. In *Arabidopsis*, *IND* specifies the identity of the lignified layer, and together with *SPT* and *ALC* promotes the formation of the separation layer (Rajani and Sundaresan, 2001; Liljegren *et al.*, 2004). However, *IND* is Brassicaceae-specific and all pre-duplication genes are more similar in sequence to its paralogue *HEC3* (Pabón-Mora *et al.*, 2014; Ortíz-Ramírez *et al.*, 2019). So far, our preliminary data indicate that *HEC3* in *Eschscholzia californica* is only restricted to early stages of flower development during the initiation of floral organs, the style, the stigma, ovules and seeds (Supplementary Data Fig. S8), but no expression is detected during fruit development, suggesting that it is perhaps more important for gynoecium patterning than for fruit development. Nevertheless, we were unsuccessful in performing *in situ* hybridization for *HEC3* in *Bocconia frutescens* and more data are required to rule out *HEC3* roles in fruit patterning.

In summary, many players are involved in the intricate fruit development and patterning genetic network (Fig. 5). All of the key transcription factors belong to different gene lineages that have duplicated independently across angiosperms but have only been functionally tested in *Arabidopsis*. Based on protein–protein interactions and expression patterns of the different genes in the wild-type and in the different *Arabidopsis* mutants, it is known that AP2 in Arabidopsis is a major negative regulator of BP and SHP (Ripoll *et al.*, 2011). Simultaneously, FUL in the valves, and RPL in the replum, repress SHP, restricting its expression to the dehiscence zone (Roeder *et al.*, 2003). Downstream SHP factors such as IND, ALC and SPT promote proper formation of the separation layer (Ferrándiz *et al.*, 2000; Liljegren *et al.*, 2004; Dinneny and Yanofsky, 2005; Alonso-Cantabrana *et al.*, 2007; Ripoll *et al.*, 2011). In Papaveraceae, FUL may be acting in concert with AP2 to promote valve identity. In the absence of SHP functional homologues, SPT/ALC and RPL genes may be controlling the dehiscence zone together with STK. Interestingly, the main function of RPL in Arabidopsis is not that of replum identity but that of repressing valve margin genes (Roeder *et al.*, 2003; Ripoll *et al.*, 2011). Expression patterns recorded in *B. frutescens* suggest that such repression was already in place in Papaveraceae (Fig. 5). Finally, our preliminary yeast two-hybrid data using *B. frutescens* proteins also show that SPT/ALC proteins can homodimerize, but cannot interact with FUL, suggesting that the mutually exclusive expression domains are not due to direct repression and that other proteins are likely to be involved (Supplementary Data Fig. S8). All protein interactions will have to be tested in the future to more accurately assess proteins that may physically come together during fruit patterning processes.

## SUPPLEMENTARY DATA

Supplementary data are available at *Annals of Botany* online and consist of the following.

Table S1: Homologues of the different gene lineages were obtained based on previous studies. Figure S1: Protein sequences of *BofrFL1*, *BofrFL2*, *BofrFL3* and *BofrAG* showing the place where the primers were designed for the probe synthesis (red box). Figure S2: Maximum likelihood tree for the *AP2/TOE3* gene lineage with extensive sampling in the Ranunculales. Figure S3: Maximum likelihood tree for the *AP1/FUL* gene lineage with extensive sampling in the Ranunculales. Figure S4: Maximum likelihood tree for the *SPT/ALC* gene lineage with extensive sampling in the Ranunculales. Figure S5: Maximum likelihood tree for the *RPL* gene lineage with extensive sampling in the Ranunculales. Figure S6: Maximum likelihood tree of the *AG/SHP/STK* gene lineage. Figure S7: Maximum likelihood tree of the *IND/HEC3* gene lineage. Figure S8: Yeast two-hybrid (Y2H) results for selected MADS-box and SPT proteins in mixed floral–fruit tissues in *Bocconia frutescens*.

mcaf079_suppl_Supplementary_Tables

mcaf079_suppl_Supplementary_Figure_S1-S8
