# Supplementary material for: Dehiscent fruits in Brassicaceae and Papaveraceae: convergent morpho-anatomical features with divergent underlying genetic mechanisms
Source: Ann Bot. 2025 May 4;136(7):1429–39. doi: 10.1093/aob/mcaf079 (PMC12718050; doi:10.1093/aob/mcaf079)
Supplement: mcaf079_suppl_Supplementary_Figure_S1-S8 [file mcaf079_suppl_supplementary_figure_s1-s8.docx]

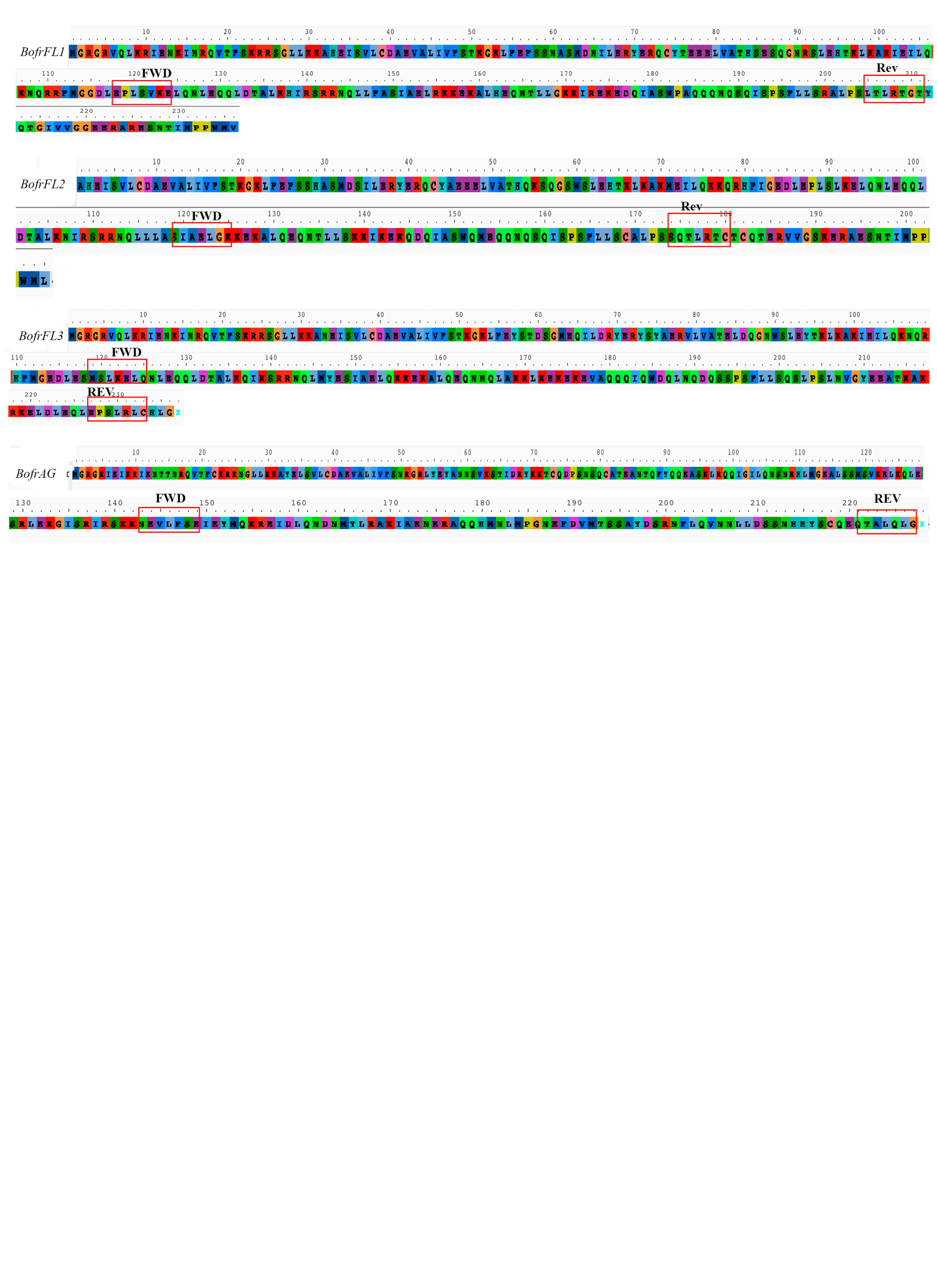
Supplemental figure 1. Protein sequences of *BofrFL1, BofrFL2, BofrFL3* and *BofrAG* showing with a red box the place where the primers were designed for the probe synthesis.


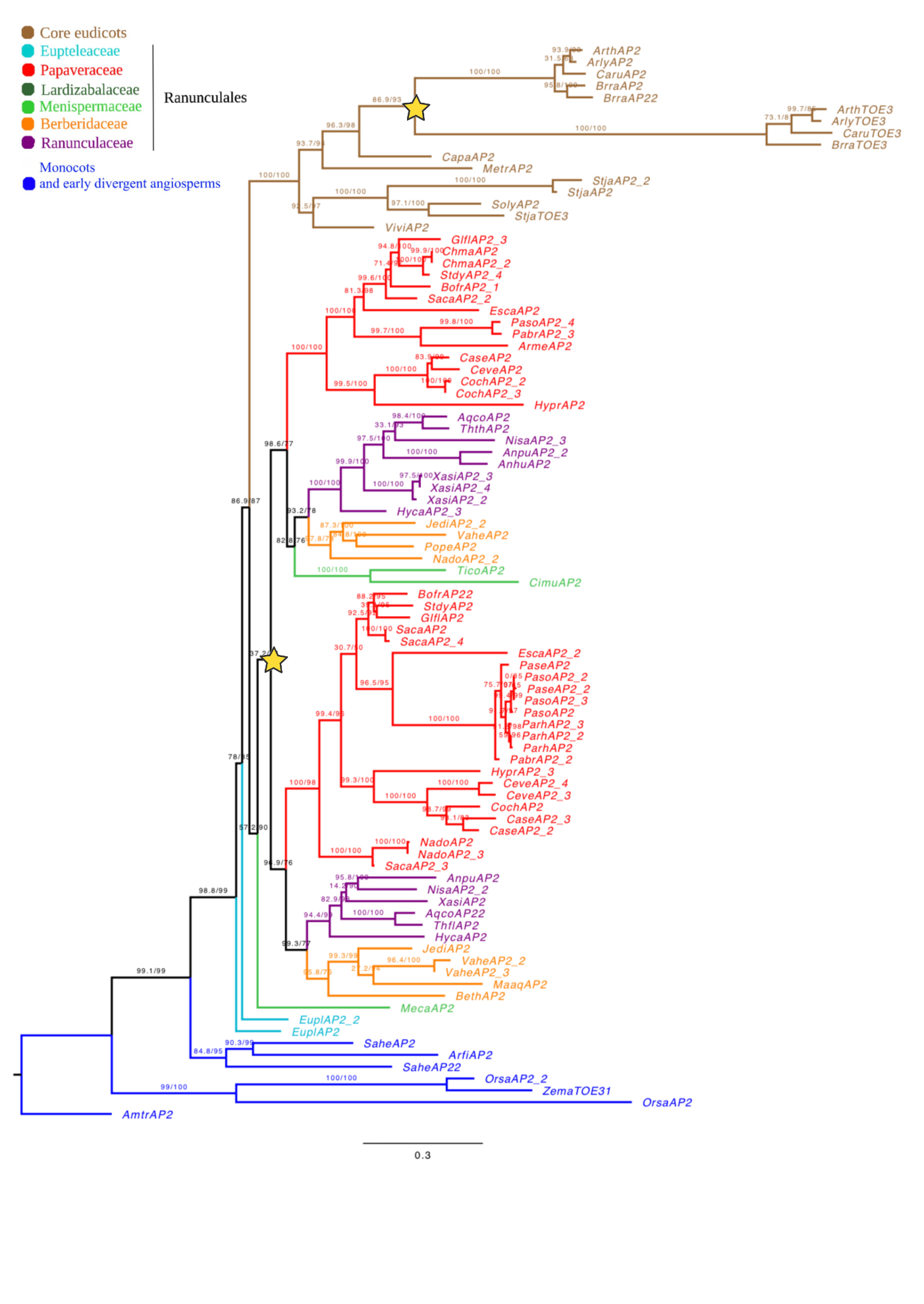


Supplemental figure 2. Maximum Likelihood for *AP2/TOE3* gene lineage with extensive sampling in the Ranunculales. Yellow stars point to two major duplication events, one in Brassicales and the other one prior to diversification of Ranunculales. Colors in the tree follow the top left convention


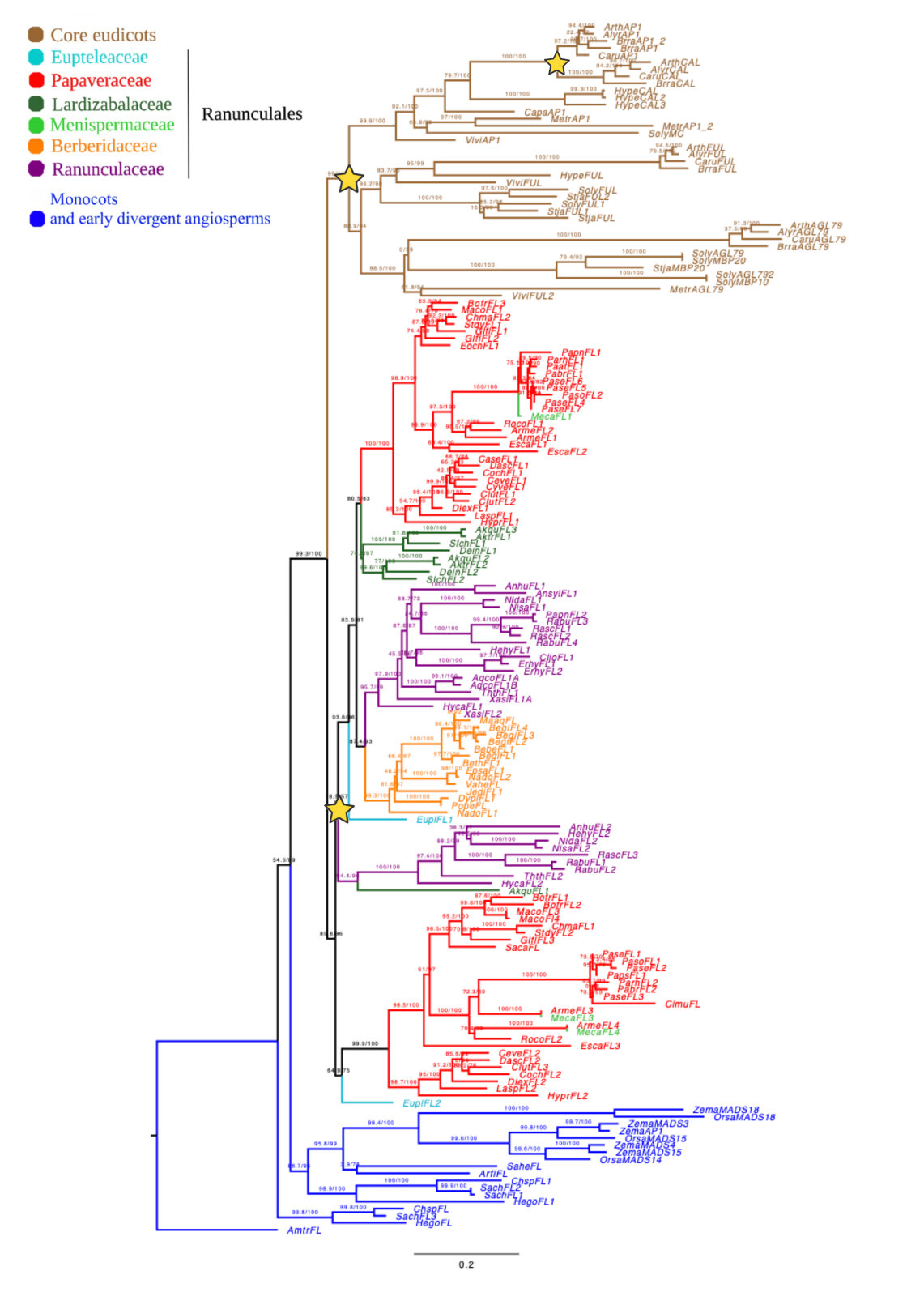


Supplemental Figure 3. Maximum Likelihood for *AP1/FUL* gene lineage with extensive sampling in the Ranunculales. Yellow stars point to two major duplication events, one in Brassicales, two prior to the diversification of core eudicots and the other one prior to diversification of Ranunculales. Colors in the tree follow the top left convention.


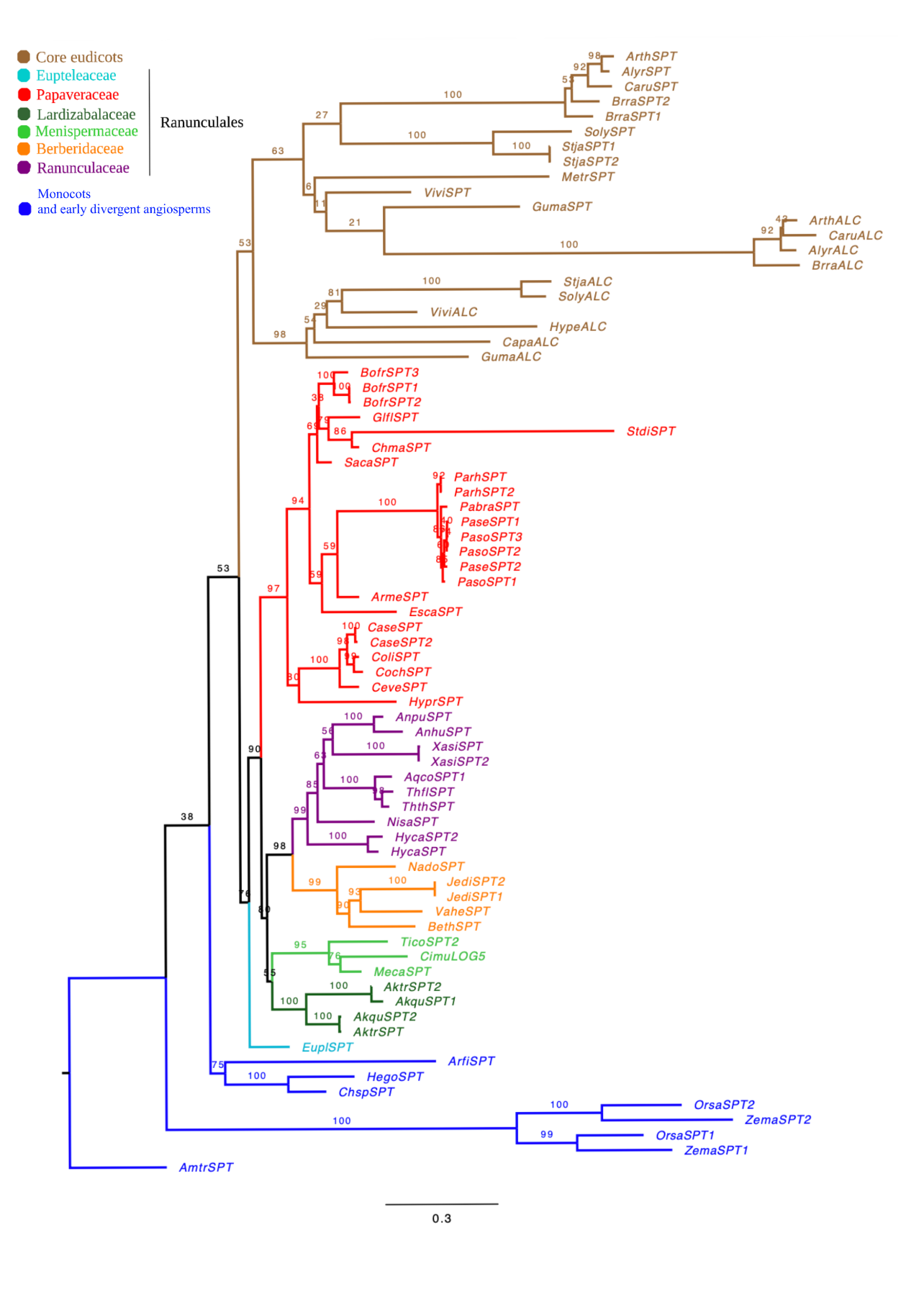


Supplemental figure 4. Maximum Likelihood for *SPT/ALC* gene lineage with extensive sampling in the Ranunculales. One duplication event has been previously identified in the core eudicots, it has not been marked here due to lack of support in this topology, possibly due to lack of sampling within this lineage. Colors in the tree follow the top left convention.


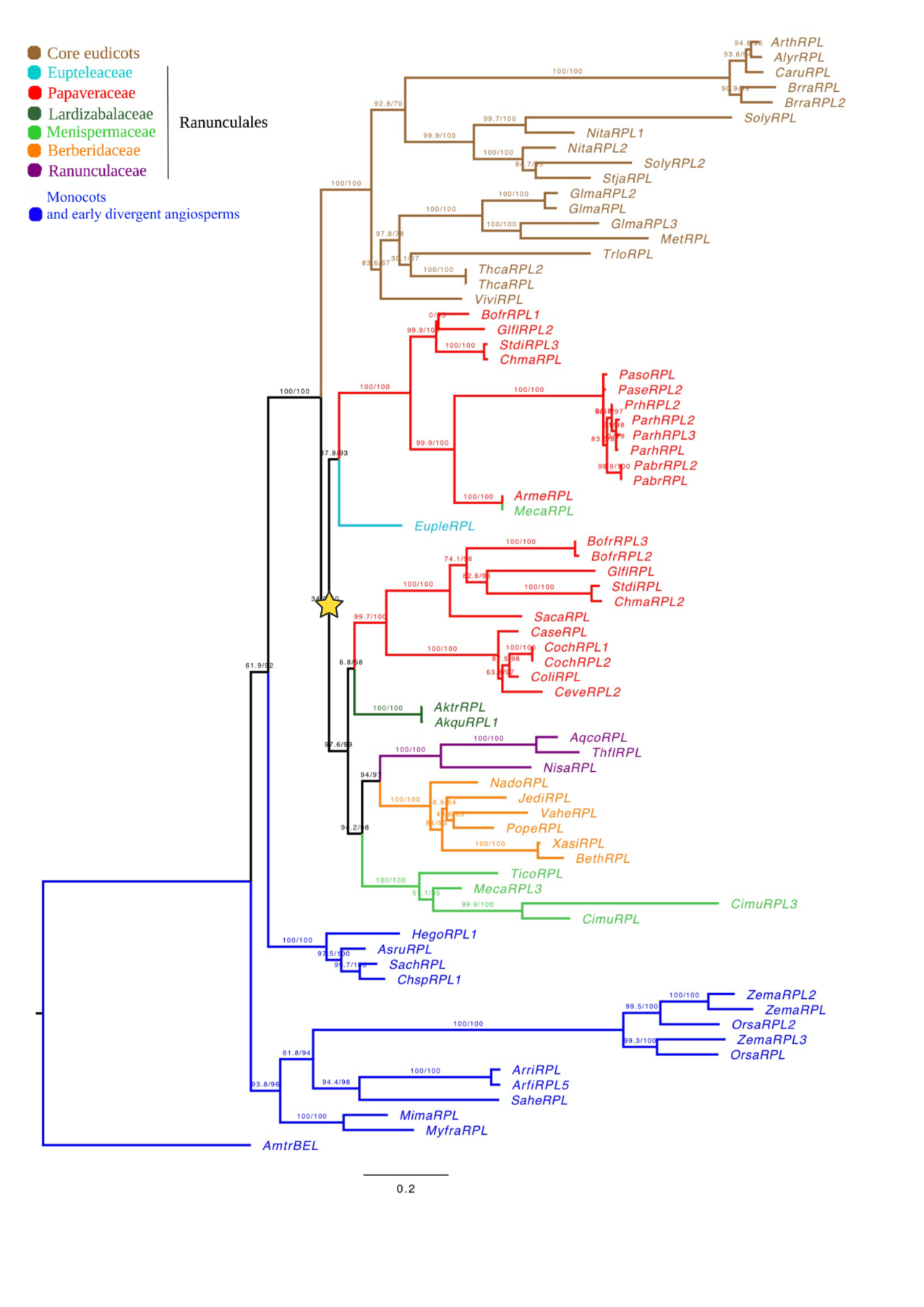


Supplemental figure 5. Maximum Likelihood for *RPL* gene lineage with extensive sampling in the Ranunculales. Yellow star point to one duplication event was identified in the Ranunculales. Colors in the tree follow the top left convention.


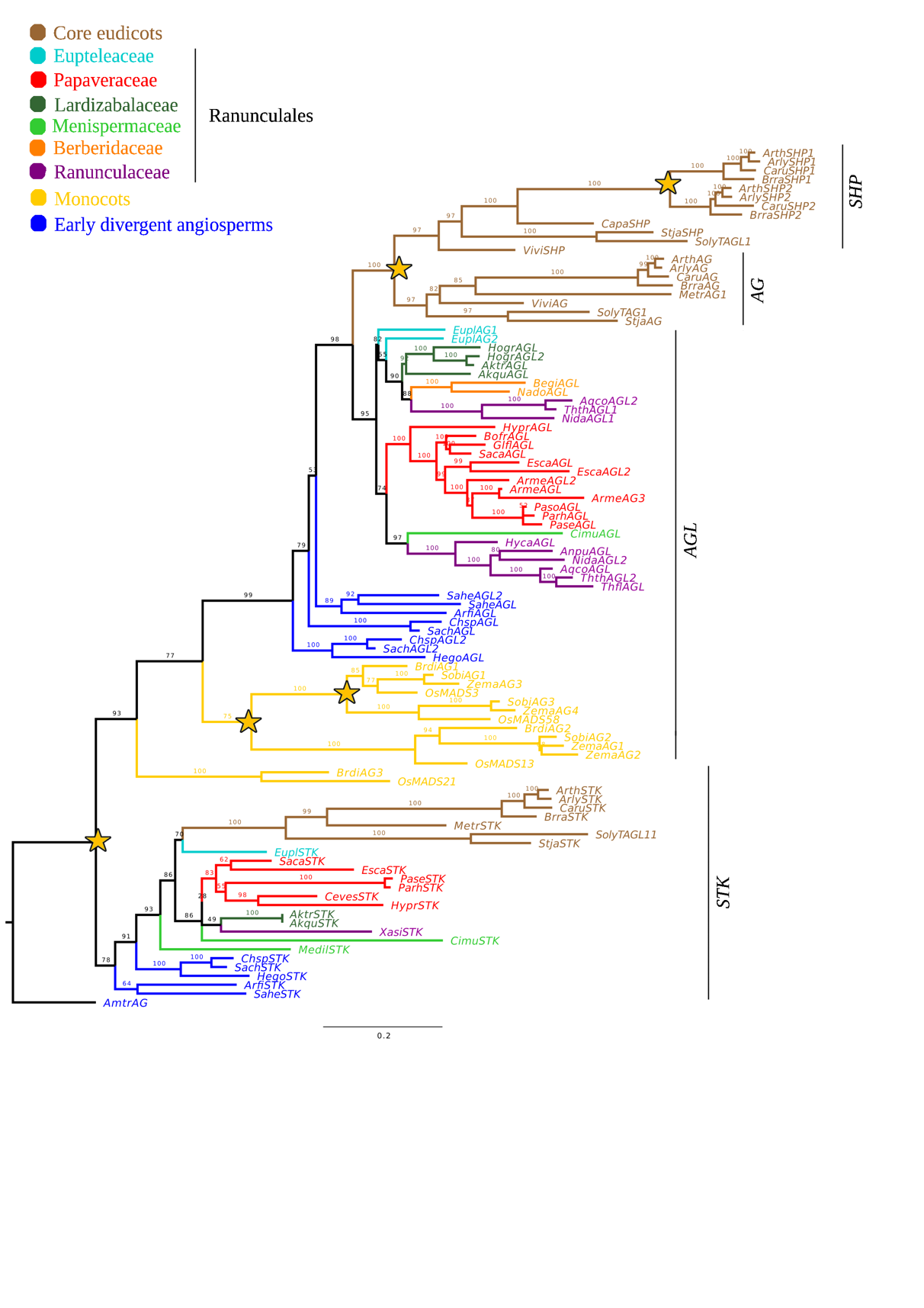


Supplemental figure 6. Maximum Likelihood of the *AG/SHP/STK* gene lineage. Yellow stars point to major duplication events, from the bottom to the top: one prior to angiosperm evolution, two in monocots, one prior to core eudicots, and one prior to Brassicaceae.


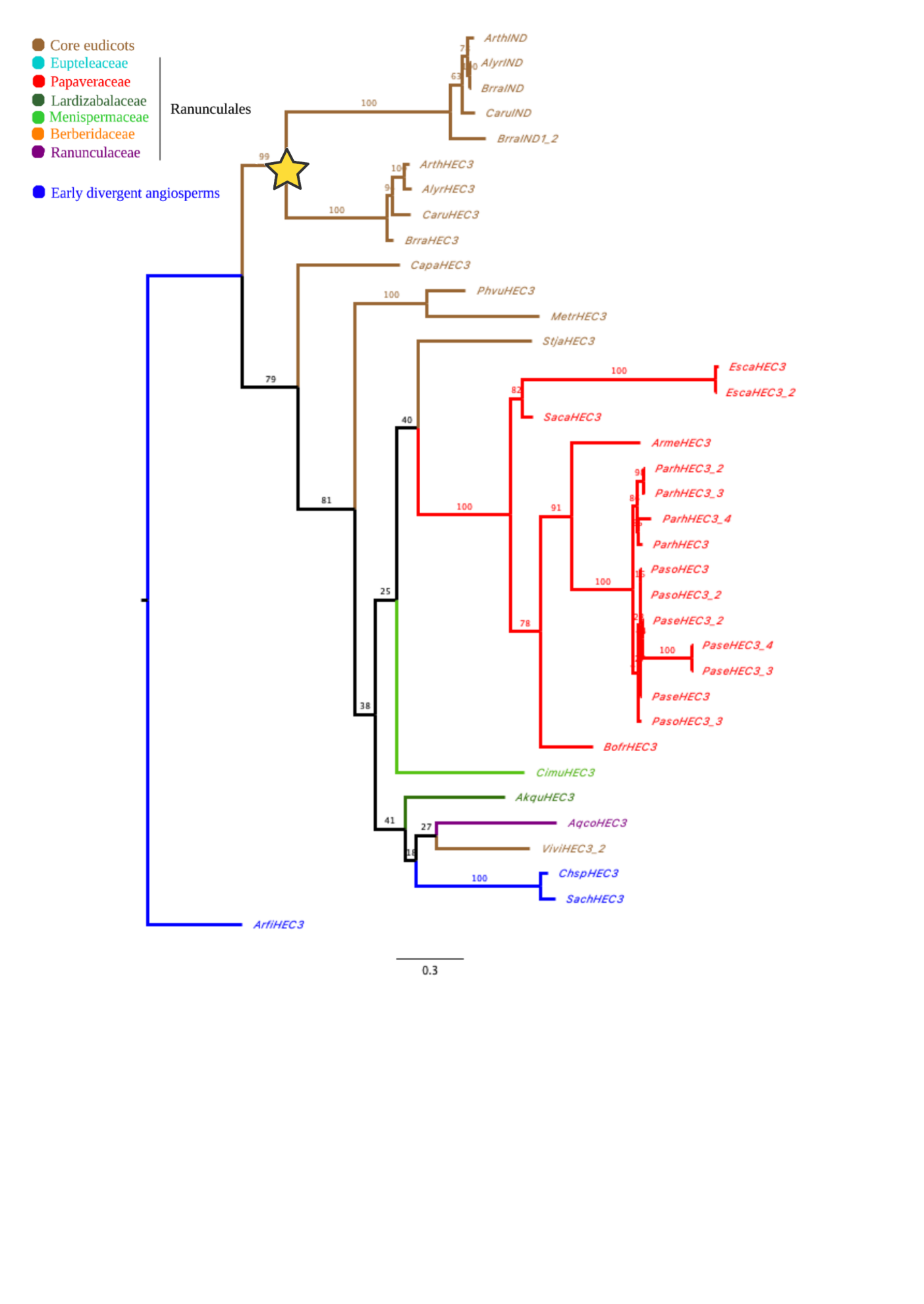


Supplemental figure 7. Maximum Likelihood of the *IND/HEC3* gene lineage. Yellow star point to a major duplication event before the diversification of Brassicales.


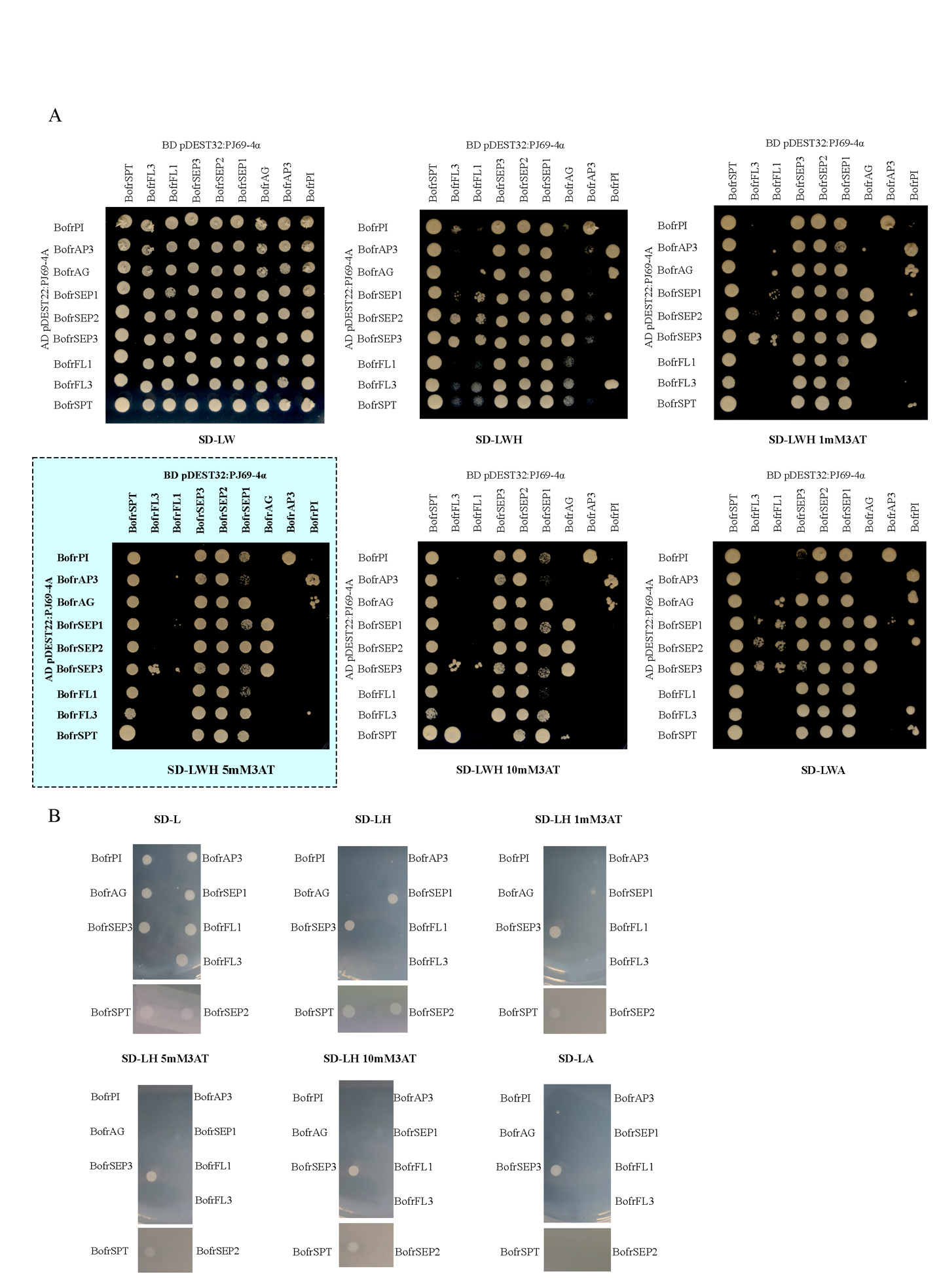


Supplemental figure 8. *Yeast two Hybrid (Y2H)* results for selected MADS-box and SPT proteins in mixed floral-fruit tissues in *Bocconia frutescens*. (A) Yeast colony growth on selective media. Potential interaction capacity was tested on SD glu medium lacking leucine (L), tryptophan (W), Histidine (H) or adenine (A), and supplemented with 1 mM, 5 mM and 10m 3AT (3-amino-1,2,4-triazole) concentration after 3 days of incubation. Blue rectangle highlights the SD -LWH 5mM3AT media selected as reference. Proteins were cloned into both activation domain (AD-pDEST22) and binding domain (BD-pDEST32) vectors and all combinations are showed. The vectors were transformed into the PJ69-4A and PJ69-4α yeast strains (Jiang, 2022; Jiang et al., 2022). Protein–protein interaction assays in yeast were performed using the GAL4 System using Gateway vectors as described (De Folter and Immink, 2011). (B) Autoactivation test of the binding domain BD-pDEST32 to define reference concentration of 3AT. BofrSPT and BofrSEP3 genes showed autoactivation even in the highest inhibitor concentration.
